# Structural and Individual Ageism Predicts Elder Abuse Proclivity and Perpetration

**DOI:** 10.1093/geroni/igab046.338

**Published:** 2021-12-17

**Authors:** E-Shien Chang, Joan Monin, Daniel Zelterman, Becca Levy

**Affiliations:** 1 Weill Cornell Medicine, New York City, New York, United States; 2 Yale School of Public Health, New Haven, Connecticut, United States; 3 Yale University, Woodbridge, Connecticut, United States

## Abstract

Considering that elder abuse affects one in six older persons worldwide, a need exists to identify factors that predict this abuse. Previous studies have found that ageism operates at both structural (i.e., societal-level stigmatizing views toward older persons) and individual levels (i.e., negative age beliefs) to affect health. However, it was not known whether and if so, how these two levels work together to impact perpetrators committing elder abuse. Thus, examining the mechanism between ageism and elder abuse was the aim of the current study. We hypothesized that structural and individual ageism would simultaneously predict elder abuse. In addition, following Stereotype Embodiment Theory, the impact of structural ageism on elder abuse would be mediated by individual ageism. In Sample 1, participants described their proclivity to abuse older people if they could do so without punishment (n=1,580). In Sample 2, family caregivers described actual abuse of their older care recipients (n=400). Overall, elder abuse proclivity (33% in Sample 1) and perpetration (56% in Sample 2) were prevalent. As hypothesized, structural ageism and individual ageism simultaneously predicted elder abuse proclivity and perpetration. Also as predicted, individual ageism significantly mediated the association between structural ageism and elder abuse in both samples. This the first study that examined the mechanistic pathways between structural and individual levels of ageism in the context of elder abuse. Effective solutions to prevent elder abuse should incorporate upstream interventions to mitigate the adverse effects of ageism.

